# MUSCLE STRENGTH AND INCIDENT DEMENTIA IN THE NATIONAL HEALTH AND AGING TRENDS STUDY (NHATS)

**DOI:** 10.1093/geroni/igad104.0546

**Published:** 2023-12-21

**Authors:** Amal Wanigatunga, Yidan Lu, Francesca Marino, Anis Davoudi, Ryan Dougherty, Jennifer Schrack

**Affiliations:** Johns Hopkins School of Public Health, Baltimore, Maryland, United States; Johns Hopkins School of Public Health, Baltimore, Maryland, United States; Johns Hopkins Bloomberg School of Public Health, Baltimore, Maryland, United States; Johns Hopkins University, Baltimore, Maryland, United States; Johns Hopkins University, Baltimore, Maryland, United States; Johns Hopkins University, Baltimore, Maryland, United States

## Abstract

Slower gait speed is linked to dementia risk, but the role muscle strength plays in this relationship is unclear. We examined whether muscle strength measured in the hands and legs is related to dementia risk beyond usual gait speed. We included 3,212 NHATS participants (mean age 75 years, 54% women) followed from 2011-2019 without dementia at baseline. Muscle strength was measured by maximal grip strength (kg) and repeated chair stands (seconds). Incident dementia combined possible and probable dementia defined by a validated NHATS algorithm. Cox regression models were used to estimate longitudinal associations between each baseline muscle strength and incident dementia. Models were adjusted for demographics and medical conditions, and additionally for usual paced gait speed (m/s). Over an average of 5 (SD=3) years, there were 946 (30%) first-time dementia events. For 1 kg higher in grip strength, there was a 2% decrease in incident dementia (HR:0.98; 95%CI: 0.97-0.99;p<0.001), which remained significant after adjustment for gait speed (p=0.005). More time to complete chair stands was positively associated with dementia incidence (HR:1.02; 95%CI:1.01-1.03;p<0.001) but results were attenuated after adjusting for gait speed (p>0.10). All results remained robust after removing events that occurred 1 year from baseline. These findings suggest that grip strength, after accounting for gait speed, is associated with dementia incidence. These results suggest the muscle morphology differences between the hands and legs capture different neuromuscular mechanisms linked to dementia. Replication of these findings and further investigation into potential biological mechanisms are needed.

